# Supplementary material for: Is socioeconomic segregation of the poor associated with higher premature mortality under the age of 60? A cross-sectional analysis of survey data in major Indian cities
Source: BMJ Open. 2018 Feb 10;8(2):e018885. doi: 10.1136/bmjopen-2017-018885 (PMC5829777; doi:10.1136/bmjopen-2017-018885)
Supplement: Supplementary data [file bmjopen-2017-018885supp001.pdf]

Appendix 1: Examining the unadjusted isolation index of socioeconomic segregation in Delhi Districts

Previous analyses on the spatial distribution of slums in Delhi has shown that the South Delhi District area has the largest concentrations of slums, while the central business district area (or New Delhi District) has the smallest concentration of slums (Ishtiyaq and Kumar 2011). South Delhi is where there exists the largest concentration of squatter settlements in Delhi, which is partly because of the proximity to high income residential neighbourhoods and commercial centres. New Delhi is the central business district of the metropolis with most of the central administrative offices. With an organized and controlled developed zone as well as lack of unorganized or unusual open spaces, this zone does not provide favourable place for squatter settlements. Other studies have also commented on the low concentration of slum households in South West Delhi areas (JNRUM 2006).

Figure 2 examines the scatterplot of the illiteracy rate and the unadjusted index of isolation for the Delhi districts estimated from the DLHS-3 data. Although there appeared to be large differences between Delhi districts in the illiteracy rate, this is largely due to the scale of the y-axis. The maximum difference in illiteracy rates between Delhi districts was around 4%, and considerably smaller than the differences between other Indian districts shown in Figure 1 of the main manuscript. South Delhi was the district where the poor are most isolated, and New Delhi and South West Delhi were the districts where the poor are least isolated. This pattern is thus consistent with previous studies on the spatial distribution of slum populations in Delhi (Ishtiyaq and Kumar 2011, JNRUM 2006).

Figure 2: Scatterplot of District Level Illiteracy Rate and Index of Isolation in 9 Delhi Districts- DLHS-3 (2008)

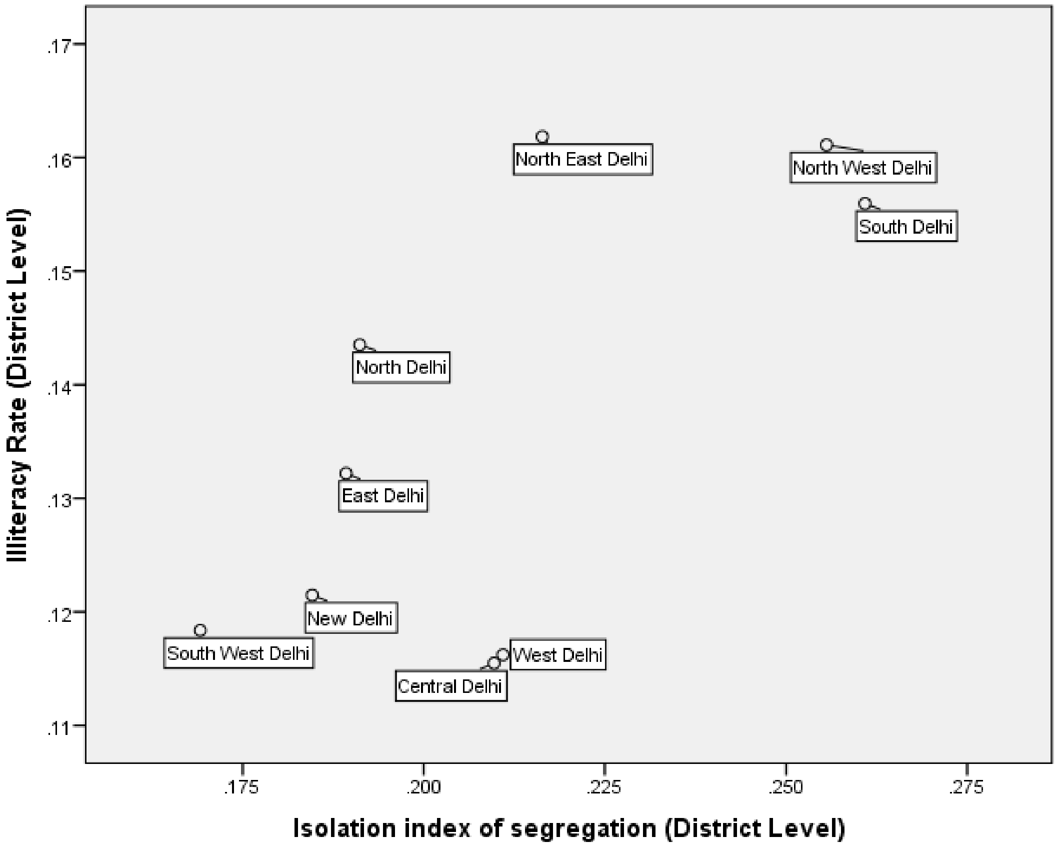

References:

Ishtiyaq, M. and S. Kumar, 2011. Typology of Informal Settlements and Distribution of Slums in the NCT, Delhi. J. Contemporary India Studies Space Soc. Hiroshima Univ., 1: 37-46 https://www.idosi.org/mejsr/mejsr7(6)11/33.pdf

JNRUM 2006. City development plan Delhi. Department of urban development. Government of IOndia. IL & FS Ecosmart Limited, New Delhi, India. http://ud.delhigovt.nic.in/CDPFull27.03.2007.pdf
