# Supplementary material for: Is socioeconomic segregation of the poor associated with higher premature mortality under the age of 60? A cross-sectional analysis of survey data in major Indian cities
Source: BMJ Open. 2018 Feb 10;8(2):e018885. doi: 10.1136/bmjopen-2017-018885 (PMC5829777; doi:10.1136/bmjopen-2017-018885)
Supplement: Author's manuscript [file bmjopen-2017-018885.draft_revisions.pdf]

BMJ Open is committed to open peer review. As part of this commitment we make the peer review history of every article we publish publicly available.

When an article is published we post the peer reviewers' comments and the authors' responses online. We also post the versions of the paper that were used during peer review. These are the versions that the peer review comments apply to.

The versions of the paper that follow are the versions that were submitted during the peer review process. They are not the versions of record or the final published versions. They should not be cited or distributed as the published version of this manuscript.

BMJ Open is an open access journal and the full, final, typeset and author-corrected version of record of the manuscript is available on our site with no access controls, subscription charges or pay-per-view fees (http://bmjopen.bmj.com).

If you have any questions on BMJ Open's open peer review process please email editorial.bmjopen@bmj.com

BMJ Open

Socioeconomic segregation of the poor is associated with higher premature mortality under the age of 60 in major Indian cities

Journal:	*BMJ Open*
Manuscript ID	bmjopen-2017-018885
Article Type:	Research
Date Submitted by the Author:	28-Jul-2017
Complete List of Authors:	Chandola, Tarani; University of Manchester, The Cathie Mash Institute and Department of Social Statistics<br>Mikkilineni, Sitamma; IFHE University, IBS<br>Chandran, Anil; University of Kerala<br>Bandyopadhyay, Souvik ; IIPH, Hyderabad<br>Zhang, Nan; University of Manchester, CMIST<br>Bassanesi, Sergio; UFRGS
**Primary Subject Heading**:	Global health
Secondary Subject Heading:	Public health
Keywords:	EPIDEMIOLOGY, SOCIAL MEDICINE, PUBLIC HEALTH

SCHOLARONE™
Manuscripts

For peer review only

Socioeconomic segregation of the poor is associated with higher premature mortality under the age of 60 in major Indian cities

Tarani Chandola, tarani.chandola@manchester.ac.uk , CMIST and Department of Social Statistics, Manchester, University of Manchester
Sitamma Mikkilineni, sitamma.m@gmail.com , IBS Business School, Hyderabad, IFHE University
Anil Chandran S, anilchandrans@gmail.com , Department of Demography and Population Studies, Thiruvananthapuram, University of Kerala
Souvik Bandyopadhyay, souvik.kumar@iiph-hydedu.in , Indian Institute of Public Health, Hyderabad, Public Health Foundation of India
Nan Zhang, nan.zhang-2@manchester.ac.uk , CMIST and Department of Social Statistics, Manchester University of Manchester
Sergio Luiz Bassanesi, sergio.bassanesi@ufrgs.br , Departamento de Medicina Social Porto Alegre, Universidade Federal do Rio Grande do Sul

Corresponding author:
Tarani Chandola <tarani.chandola@manchester.ac.uk>
CMIST and Department of Social Statistics,
HBS Building,
University of Manchester
Manchester M13 9PL
Tel: +44 161 306 6903
Fax: +44 161 275 4722

Keywords: urban health, social segregation, socioeconomic factors, premature mortality
Word count: 2844
Reference count: 22

For peer review only - http://bmjopen.bmj.com/site/about/guidelines.xhtml

ABSTRACT

Objectives: Although urbanisation is generally associated with poverty reduction in developing counties, in some contexts, it results in increased socioeconomic segregation of the poor. The association of socioeconomic segregation with higher mortality is based on weak evidence. The paper examines whether socioeconomic segregation of the poor is associated with higher under-60 years ("premature") mortality risk in Indian cities and whether this association is confounded by sociodemographic and socioeconomic factors.

Setting and participants: A population representative sample from 39,427 households living in 1,876 urban wards within 59 Indian districts (cities) with more than a population of 1 million from the third (2008) District Level Household Survey.

Primary outcome and other measures: The outcome was any death under the age of 60 reported by households in the preceding 4 years of the DLHS-3 survey. An index of socioeconomic segregation (an isolation index of the poor) was estimated at the district level.

Results: The Index of isolation was associated with premature mortality, even after taking into account socio-demographic and socioeconomic confounders. A unit increase in the isolation index was associated with an increase of 0.66 (95%CI: 0.15-1.17) in the probit of premature mortality which is an absolute increase in premature mortality of 9%. Moreover, poor people who live in more socioeconomically segregated neighbourhoods had higher probabilities of premature death than poor people living in less segregated neighbourhoods.

Conclusion: Increasing segregation of the poor may result in higher premature mortality. As developing countries become increasingly urbanised, there is a risk that this leads to increased segregation of the poor as well as increased premature mortality.

For peer review only - http://bmjopen.bmj.com/site/about/guidelines.xhtml

Strengths and limitations

- The study examines how residential socioeconomic segregation is related to premature mortality.
- This is an important topic because urbanisation in developing countries is often accompanied by an increasing spatial concentration of poverty in cities.
- The increased risk of premature mortality associated with urban socioeconomic segregation remains even after taking into account household levels socioeconomic and sociodemographic confounders.
- This is a cross-sectional study and we have not observed whether increasing socioeconomic segregation results in higher premature mortality.
- We have not estimated any lagged effects of socioeconomic segregation on premature mortality

For peer review only

For peer review only - http://bmjopen.bmj.com/site/about/guidelines.xhtml

INTRODUCTION

Urbanisation in developing countries is often accompanied by an increasing spatial concentration of poverty in cities. Although urbanisation can play a positive role in overall poverty reduction, when it is accompanied by weak economic growth or when distributive policies are non-existent or ineffective, it results in the socioeconomic segregation of poor people through the concentration of poor people living in poor localities, rather than significant poverty reduction [1]. Although levels of urbanisation in India are relatively low at around 31 percent in 2011, the process of urbanisation is among the fastest in the world. As a result of urbanisation, India contains the highest number of urban slum dwellers, accounting for 17 per cent of the world's slum dwellers [1].

The association between poverty and ill health is well documented. The risk of premature mortality and disease as a result of living in poor areas such as slums with inadequate housing, lack of access to safe water and adequate sanitation, is well known. Furthermore, there are now a multitude of studies showing associations between area level poverty rates and deprivation and poor health/mortality, even after taking into account individual and household level socioeconomic circumstances [2-4]. This suggests an area or contextual effect on health associated with living in a poor area, over and above the compositional effect of the number of poor people in an area. What is perhaps less well known is the association between socioeconomic segregation and health. Studies have shown that there are ecological correlations between socioeconomic segregation by income and mortality rates [5-8]. These studies have shown that greater socioeconomic segregation of the poor within cities is associated with higher mortality rates. However, there are few, if any analyses that take into account potential confounders of this ecological correlation, such as the strong association between poverty and ill health among individuals and families. It is likely that as people from poor households live in poor areas that are socioeconomically isolated from the rest of the city, any association between socioeconomic segregation and health needs to take account of such confounding from socioeconomic factors at the compositional (household or individual) level. Furthermore, it is likely that socioeconomically segregated poor people have poorer health than other poor people who are living in less segregated areas. . The concept of a 'triple health jeopardy' [5] of being poor, living in a poor district that is socioeconomically segregated from the rest of the city has been suggested, but there has been little empirical evidence linking individual mortality records to household poverty and area level segregation.

The main research question of this paper is to investigate whether being poor, living in a poor area that is socioeconomically isolated from the rest of the city is associated with greater mortality risk compared to poor people living in less socioeconomically segregated areas.

For peer review only - http://bmjopen.bmj.com/site/about/guidelines.xhtml

METHODS

Datasets

Data from the third District Level Household Survey [9] were analysed, with the survey conducted between 2007 to 2008. The District Level Household Survey was designed to provide information on reproductive and child health in all the districts of India. The data are made available on request through the website http://rchiips.org/obtainingdata.html. The steps involved in the sampling of households were:

(i) the selection of primary sampling units (PSUs) within census wards;

(ii) the selection of the households from each of the selected village/urban PSUs through a random selection of households within a census enumeration block (which we have used to define as a neighbourhood in this study).

The DLHS-3 PSUs were made available for secondary data analyses, allowing the estimating of neighbourhood socio-economic segregation indices.

For this study, the analytical sample was restricted to households living within urban wards of the largest cities with a population of more than 1 million. This resulted in an analytical sample of 39,446 households living in 1,876 urban wards within 59 cities. This was to ensure comparability of the urban units within the analysis. Mega cities such as Kolkata, Mumbai and Delhi were disaggregated into their constituent administrative districts.

Outcome

Premature mortality under the age 60 was reported for each household participating in the DLHS-3 in the preceding four years (from 2004-2008). This was recoded into a binary variable (any premature death in the household vs. no premature death) for the probit regression analysis. The under-60 death rate was calculated at the district level by dividing the total premature mortality in a district by the number of respondents aged under-60 in the DLHS-3 sample.

The Isolation Index of Segregation

Although there are a number of measures of segregation, the isolation index of segregation was used in this study as socioeconomic isolation is a key feature of the research question. The study used estimates of neighbourhood socio-economic composition from DLHS-3 districts and primary sample units (PSUs). PSUs correspond to around 300 households from a census enumeration block and we used them to represent urban “neighbourhoods”. Poverty was measured through the proxy indicator of illiteracy as direct measures of poverty were not available in the data. The index of isolation was used to measure the socioeconomic segregation of the poor. The isolation index (Ind_isolation_j) measures the extent to which poor members of a district are exposed only to one another and is computed as the weighted average of the proportion of poor people in each neighbourhood [10].

For peer review only - http://bmjopen.bmj.com/site/about/guidelines.xhtml

$$\text{Ind\_isolation}_j = \sum_{i=1}^{j} \frac{\text{illiterate}_{ij}}{\text{illiterate}_j} \times \frac{\text{illiterate}_{ij}}{\text{population}_{ij}}$$

where

illiterate_{ij} is the number of illiterate adults living in neighbourhood_i within district_i

Illiterate_j is the number of illiterate adults living in district_j

Population_{ij} is the adult population living in neighbourhood_i within district_i

The DLHS-3 survey weights were used to derive the district and neighbourhood level estimates of the illiterate and total populations. As the DLHS-3 does not contain information on all neighbourhoods within a district, the estimated Index may not accurately reflect the true distribution of the district level Isolation Index. However, the use of the DLHS-3 survey data does allow for an estimate of the Index at the neighbourhood level (through the random sample of households within a primary sampling unit), rather than estimating the Index at larger census area levels such as the ward level, which often refers to a very large area and population (around 25,000-75,000) for the largest Indian cities.

The Index can vary from 0 to 1 where higher values denote greater segregation of the poor (measured using illiteracy as a proxy). An interpretation of the index is the probability that a poor person will live next to another poor person within the neighbourhood.

Covariates

As there are no direct measures of income or poverty in the DLHS data, poverty was measured through the use of a proxy indicator namely illiteracy. Another reason for using illiteracy to measure poverty is the difference in the meanings and concepts of standard poverty measures (such as indicators of wealth and status) across Indian cities. The illiteracy rates were calculated by dividing the total number of illiterate adults in a DLHS-3 household or district by the number of adults in that household or district. Other potential sociodemographic and socioeconomic confounders included the number of household members, household wealth (constructed by the DLHS-3 survey team from a factor analysis of consumption measures and housing quality indicators), and religion of head of household, whether the head of household belonged to a scheduled caste (SC) or scheduled tribe (ST), and whether the household accessed government or private health care when someone was ill.

Analysis

Scatterplots were used to examine the ecological correlation between the district level Index of isolation of the poor, the district level illiteracy rate and the district level under-60 mortality rate. Multilevel probit regression (with the outcome of under-60 death within a household) was used to estimate the effect of the

For peer review only - http://bmjopen.bmj.com/site/about/guidelines.xhtml

(district level) Index of isolation, after taking into account socioeconomic and sociodemographic factors at the household levels. Multilevel analysis is particularly appropriate as it takes into account the sample design of the DLHS-3 which results in clustered samples at the state, district and neighbourhood (PSU) levels- each of these levels were specified with a random intercept in the multilevel models. It is important to take this clustering into account in the analysis as the main exposure variable is at the district level, rather than at the household level. The multilevel regression coefficients were estimated using the PQL2 estimation procedures within MLwin.

The question of whether being poor, living in a poor area that is socioeconomically segregated from the rest of the city is associated with greater mortality risk, was examined through an interaction between the household illiteracy rate ("being poor") with the isolation index ("living in a poor area that is socioeconomically segregated"). For comparison, the interaction between the household illiteracy rate and the district level illiteracy rates were also examined. As the proportions of missing data were very low, complete case analyses were performed.

RESULTS

The scatterplot of the district level associations between premature death rates and the Index of Isolation (Figure 1) shows that there was a moderate ecological correlation (R=0.52). Districts (cities) where the poor were more isolated were also those with higher premature death rates. Only a few of the 59 districts are displayed in the scatterplot to aid ease of interpretation. Some of the districts such as North West Delhi and West Delhi had the same levels of segregation, but differed in terms of premature death rates by more than 5%. The ecological correlation between premature death rates and the District level illiteracy rates was a little weaker (r=0.49). The ecological correlation (for all the 59 districts) between the index of isolation and illiteracy rate was very strong (r=0.90) which means that using both in a regression analysis is likely to lead to multicollinearity problems.

Table 1 displays the distribution of the key independent variables in the analysis by any premature death in the household. 5.3% of all households in the analytical sample reported the death of a household member aged under 60 in the four years period preceding the survey. Households where there was a premature death in the preceding 4 years were living in districts with higher Index of isolation scores, higher illiteracy rates at the district and household levels, lower wealth scores, more household members, were more likely to be Muslim and Scheduled Caste/Scheduled Tribe households and also were more likely to use government clinics when ill.

For peer review only - http://bmjopen.bmj.com/site/about/guidelines.xhtml

Table 1 Distribution (Mean and Percentages) of key independent variables in the analysis by premature death (under 60) in the household: DLHS-3

	No death under 60 in the household	≥1 death under 60 in the household
n	*37346*	*2100*
Isolation Index (District level)	0.22 (0.06)	0.23 (0.06)
Illiteracy rate (District level)	0.15 (0.05)	0.16 (0.05)
Illiteracy rate (Household level)	0.16 (0.24)	0.23 (0.29)
Mean wealth index score	1.33 (0.93)	1.05 (0.92)
Mean number of household members	4.92 (2.37)	5.25 (2.78)
% of households that are Muslim	16.4%	19.3%
% of households that are SC/ST	18.4%	24.1%
% of households using government clinics when ill	38.4%	41.9%

SC: Scheduled Caste; ST: Scheduled Tribe; DLHS: District Level Household Survey.

Table 2 displays the results (estimated probits and 95% confidence intervals) of the multilevel probit regression models with any premature death in the household as the dependent variable. In Model 1, the model with all the household level sociodemographic and socioeconomic factors was fitted, taking into account the clustering in premature mortality at the state, district, primary sampling unit (PSU) and household levels. The probit model constrains the variance at the lowest (household) level to be one, and there appeared to be some clustering in premature mortality at the PSU and district levels, but a negligible amount of clustering at the state level. Being a member of an illiterate or poorer household with more household members, and living in the Northern region were associated with increased probabilities of premature mortality. Model 2 added in the Isolation Index as an independent variable. A unit increase in the isolation index (going from 0 to 1) was associated with an increase of 0.66 in the probit of premature mortality. When converted into probabilities, the probability of premature mortality for households living in districts with no socioeconomic segregation (Index of isolation=0) was 0.04, while the probability of premature mortality for households living in districts that were completely segregated (Index of isolation=1) was 0.13. So going from no segregation to complete segregation was associated with a predicted absolute increase in premature mortality of 9%.

Model 3 added in the interaction term between the Index of isolation and the household illiteracy rate. There was some evidence that illiterate households which lived in more socioeconomically segregated neighbourhoods had a higher probability of premature death compared to illiterate households which lived in less segregated neighbourhoods. The estimated probabilities are graphed in Figure 2 which shows overlapping probabilities of premature death for illiterate and literate households living in less socio-

For peer review only - http://bmjopen.bmj.com/site/about/guidelines.xhtml

economically segregated neighbourhoods. However, as socioeconomic segregation increased, the probabilities of premature death for literate households increased only a little, whereas the corresponding probabilities for illiterate households increased greatly.

Model 4 examines the association of the district level illiteracy rates with premature mortality. A one unit increase in the district level illiteracy rate (or in other words, going from 0% to 100% illiteracy in the district) was associated with an increase of 0.73 in the probit of premature death. However, the interaction between district level illiteracy and household illiteracy was not significant (Model 5).

For peer review only

For peer review only - http://bmjopen.bmj.com/site/about/guidelines.xhtml

Table 2 Probits (and 95% confidence intervals) from Multilevel Probit Regression Models of premature mortality: DLHS-3

Fixed Part	**Model 1**	**Model 2**	**Model 3**	**Model 4**	**Model 5**
Intercept	**-1.55 (-1.62,-1.47)**	**-1.56 (-1.63,-1.49)**	**-1.56 (-1.64,-1.49)**	**-1.56 (-1.64,-1.49)**	**-1.56 (-1.64,-1.49)**
Household Illiteracy rate (ref: all hh members are literate)					
Some literate	**0.10 (0.05,0.15)**	**0.09 (0.04,0.14)**	**0.10 (0.04,0.15)**	**0.09 (0.04,0.14)**	**0.09 (0.04,0.14)**
All illiterate	**0.21 (0.10,0.31)**	**0.20 (0.09,0.31)**	**0.15 (0.03,0.27)**	**0.20 (0.09,0.31)**	**0.16 (0.04,0.28)**
Wealth tertiles (ref: poorest tertile)					
Middle wealth tertile	**-0.11 (-0.16,-0.05)**	**-0.10 (-0.16,-0.05)**	**-0.10 (-0.16,-0.05)**	**-0.10 (-0.16,-0.05)**	**-0.10 (-0.16,-0.05)**
Richest tertile	**-0.25 (-0.31,-0.19)**	**-0.25 (-0.31,-0.19)**	**-0.25 (-0.31,-0.18)**	**-0.25 (-0.31,-0.18)**	**-0.25 (-0.31,-0.18)**
Region (ref: North)					
East	**-0.17 (-0.27,-0.06)**	**-0.17 (-0.26,-0.07)**	**-0.16 (-0.26,-0.06)**	**-0.16 (-0.26,-0.06)**	**-0.16 (-0.26,-0.06)**
West	**-0.16 (-0.24,-0.08)**	**-0.13 (-0.21,-0.05)**	**-0.13 (-0.21,-0.05)**	**-0.13 (-0.21,-0.04)**	**-0.13 (-0.21,-0.04)**
South	**-0.27 (-0.36,-0.19)**	**-0.24 (-0.32,-0.15)**	**-0.24 (-0.32,-0.15)**	**-0.25 (-0.33,-0.16)**	**-0.25 (-0.33,-0.16)**
Religion (ref: Hindu)					
Muslim	0.02 (-0.04,0.08)	0.02 (-0.05,0.08)	0.01 (-0.05,0.07)	0.01 (-0.05,0.08)	0.01 (-0.05,0.07)
Other religion	0.02 (-0.07,0.10)	0.02 (-0.07,0.11)	0.02 (-0.07,0.11)	0.03 (-0.06,0.11)	0.02 (-0.06,0.11)
Caste (ref: other caste)					
SCST caste hh	**0.12 (0.06,0.18)**	**0.12 (0.06,0.18)**	**0.12 (0.06,0.18)**	**0.12 (0.06,0.18)**	**0.12 (0.06,0.18)**
Other backward caste hh	0.05 (-0.01,0.10)	0.05 (0.00,0.1)	0.05 (-0.01,0.10)	0.05 (0.00,0.10)	0.05 (-0.01,0.10)
Missing/DK caste hh	-0.05 (-0.23,0.12)	-0.05 (-0.23,0.13)	-0.05 (-0.23,0.13)	-0.05 (-0.23,0.13)	-0.05 (-0.22,0.13)
Access to private health care (ref: yes)					
Use govt clinic when ill	0.02 (-0.02,0.07)	0.02 (-0.02,0.07)	0.02 (-0.02,0.07)	0.02 (-0.02,0.07)	0.02 (-0.02,0.07)
Number of people in hh	**0.01 (0.00,0.02)**	**0.01 (0.00,0.02)**	**0.01 (0.00,0.02)**	**0.01 (0.00,0.02)**	**0.01 (0.00,0.02)**
Isolation index (District)		**0.66 (0.15,1.17)**	0.43 (-0.20,1.05)		
Interaction of Isolation Index (District) and hh Illiteracy rate					
Isolation Index*Some hh members are literate			0.32 (-0.45,1.09)		
Isolation Index*All hh members are illiterate			**2.08 (0.31,3.85)**		
Illiteracy rate (District)				**0.73 (0.11,1.35)**	0.58 (-0.18,1.33)
Interaction of Illiteracy rate (District) and hh Illiteracy rate					
Illiteracy rate (District level)*Some hh members are literate					0.17 (-0.73,1.07)
Illiteracy rate (District level)*All hh members are illiterate					1.70 (-0.41,3.81)
Random Part					
State variance (n=19)	0	0	0	0	0
District variance (n=59)	0.006	0.004	0.004	0.004	0.005
PSU variance (n=1876)	0.022	0.022	0.022	0.022	0.022
Household variance (n=39427)	1	1	1	1	1

DLHS: District Level Household Survey; SC: scheduled Caste; ST: Scheduled Tribe.

DISCUSSION

This study found that urban Indian districts where the poor were segregated (i.e. isolated) had higher premature mortality rates than districts where the poor were less segregated. Going from no socio-economic segregation to complete segregation was associated with a 9% absolute increase in the probability of premature death rate in a household in the previous 4 years. This association appeared to remain even after taking into account a range of compositional (household level) socio-demographic and socio-economic factors that could confound this association. There thus appears to be some evidence of a 'triple health jeopardy' [5] of being poor, living in a poor district that is socioeconomically segregated from

For peer review only - http://bmjopen.bmj.com/site/about/guidelines.xhtml

the rest of the city. Poor people who live in more socioeconomically segregated neighbourhoods have higher probabilities of premature death than poor people living in less segregated neighbourhoods.

While the analyses could not directly compare the Index of isolation with the district level illiteracy index as both measures were very highly correlated, there were differences in their associations with premature mortality. There was no evidence that living in a poor district was a risk factor especially for poor households, whereas living in a socioeconomically segregated district appeared to increase the risk of premature mortality particularly for poor households.

There may be a number of reasons for the relationship between socioeconomic segregation and premature mortality. Poor nutrition, sub-standard housing conditions and overcrowding increase susceptibility and exposure to infectious diseases [11-14]. Poor segregated areas, in spite of their greatest needs, often have inadequate services and restricted access to primary care services [5, 7, 15]. Poor neighbourhoods are more likely to be exposed to environmental pollutants [16-19]. Socioeconomic segregation may enable the affluent access to the greatest resources and reinforce the social exclusion of the disadvantaged [10, 20, 21]. The overall effect of income segregation appears to reinforce social inequality [22] through segregating the poor away from resources such as employment, transport, education, health care and other services, a process which could discourage social mobility. It may thus capture dimensions of social inequality distinct from poverty or deprivation rates that have typically been used to measure social inequality at an area level.

There are a number of limitations to this study. This is a cross-sectional study showing associations between premature mortality and a single measure of socioeconomic segregation. We cannot infer causality from observational evidence as we have not observed whether increasing socioeconomic segregation results in higher premature mortality. While we have adjusted for a range of potential socio-economic confounders at the district and household levels, there may be other unobserved factors that cause the association of socioeconomic segregation with premature mortality. In addition, we have not estimated any lagged effects of socioeconomic segregation on premature mortality. If the association is causal, we do not know what duration of exposure to a socioeconomically segregated neighbourhood is needed to generate higher premature mortality risks. The measure of socioeconomic segregation was estimated from survey data and so may not be accurately estimated. Appendix 1 examines the association of the index of isolation with the district level illiteracy rates for the Delhi districts, where there have been some studies on the spatial distribution of slum housing. The analysis confirms the expected ranking of Delhi districts by socio-economic segregation, suggesting that the estimated segregation index may be valid. Other dimensions of socioeconomic segregation could not be measured due to data limitations. In particular, we have not been able to measure the spatial dimension of socio-economic segregation which may be particularly relevant in relation to discussions of social inequalities.

For peer review only - http://bmjopen.bmj.com/site/about/guidelines.xhtml

As urbanisation increases in India, there is a risk of increasing socioeconomic segregation of the poor and associated health risks. Indian cities already contain the largest concentration of the world's population living in slums. With increasing urbanisation and associated economic pressures, the poor living within Indian cities are at risk of increasing isolation such as through slum resettlement programs far from employment, transport and public services networks. The results from this study cautions against such policies that increase the socioeconomic segregation of the poor, as this may lead to increased levels of premature mortality among the most vulnerable groups in society.

Contributors: TC, SB and SM contributed to the conception and design of the study. AC, SB and NZ contributed to the analysis and interpretation of the data. TC drafted the initial manuscript and SB, SM, SB, AC and NZ contributed to revisions of the manuscript. All authors gave approval for the final version and agree to be accountable for all aspects of the study.

Funding This work was supported by the Economic and Social Research Council (ESRC): RES-238-25-0015 and ES/J019119/1

Competing interests: None declared

For peer review only

REFERENCES

1 Un-habitat. *State of the world's cities 2010/2011: bridging the urban divide*. London: Earthscan 2010.
2 Van Ham M, Manley D, Bailey N*, et al. Understanding neighbourhood dynamics: New insights for neighbourhood effects research*. London: Springer 2012.
3 Pickett KE, Pearl M. Multilevel analyses of neighbourhood socioeconomic context and health outcomes: a critical review. *Journal of Epidemiology and Community Health* 2001;**55**:111-22.
4 Kawachi I, Berkman LF. *Neighborhoods and health*: Oxford University Press 2003.
5 Ross NA, Nobrega K, Dunn J. Income segregation, income inequality and mortality in North American metropolitan areas. *GeoJournal* 2001;**53**:117-24.
6 Waitzman NJ, Smith KR. Separate but lethal: the effects of economic segregation on mortality in metropolitan America. *Milbank Quarterly* 1998;**76**:341-73.
7 Lobmayer P, Wilkinson RG. Inequality, residential segregation by income, and mortality in US cities. *Journal of Epidemiology and Community Health* 2002;**56**:183-7.
8 Szwarcwald CL, de Andrade CLT, Bastos FI. Income inequality, residential poverty clustering and infant mortality: a study in Rio de Janeiro, Brazil. *Social science & medicine* 2002;**55**:2083-92.
9 International Institute for Population Sciences (IIPS). *District Level Household and Facility Survey (DLHS-3), 2007-08: India*. Mumbai: IIPS 2010.
10 Massey DS, Denton NA. The dimensions of residential segregation. *Social forces* 1988;**67**:281-315.
11 Walker SP, Wachs TD, Gardner JM*, et al.* Child development: risk factors for adverse outcomes in developing countries. *The lancet* 2007;**369**:145-57.
12 Immink MDC. The urban poor and household food security: policy and project lessons of how governments and the urban poor attempt to deal with household food insecurity, poor health and malnutrition. Concepts, evidence and case studies New York: United Nations Children's Fund (UNICEF) 1994.
13 Alirol E, Getaz L, Stoll B*, et al.* Urbanisation and infectious diseases in a globalised world. *The Lancet infectious diseases* 2011;**11**:131-41.
14 Galobardes B, Lynch JW, Smith GD. Childhood socioeconomic circumstances and cause-specific mortality in adulthood: systematic review and interpretation. *Epidemiologic reviews* 2004;**26**:7-21.
15 Hassing C, Twickler M, Brunekreef B*, et al.* Particulate air pollution, coronary heart disease and individual risk assessment: a general overview. *European Journal of Cardiovascular Prevention & Rehabilitation* 2009;**16**:10-5.
16 Anderton DL, Anderson AB, Rossi PH*, et al.* Hazardous Waste Facilities" Environmental Equity" Issues in Metropolitan Areas. *Evaluation Review* 1994;**18**:123-40.
17 Ellen IG, Mijanovich T, Dillman KN. Neighborhood effects on health: exploring the links and assessing the evidence. *Journal of Urban Affairs* 2001;**23**:391-408.
18 Cohen DA, Mason K, Bedimo A*, et al.* Neighborhood physical conditions and health. *American Journal of Public Health* 2003;**93**:467-71.
19 Ash M, Fetter TR. Who Lives on the Wrong Side of the Environmental Tracks? Evidence from the EPA's Risk ‐ Screening Environmental Indicators Model*. *Social Science Quarterly* 2004;**85**:441-62.
20 Massey DS. The age of extremes: Concentrated affluence and poverty in the twenty-first century. *Demography* 1996;**33**:395-412.
21 Jargowsky PA. Take the money and run: Economic segregation in US metropolitan areas. *American sociological review* 1996:984-98.
22 Dwyer RE. Poverty, prosperity, and place: the shape of class segregation in the age of extremes. *Social Problems* 2010;**57**:114-37.

For peer review only - http://bmjopen.bmj.com/site/about/guidelines.xhtml

Figure 1: Scatterplot of District Level Under 60 death rate and Index of Isolation in 59 Indian urban districts-DLHS-3 (2008): selected districts shown

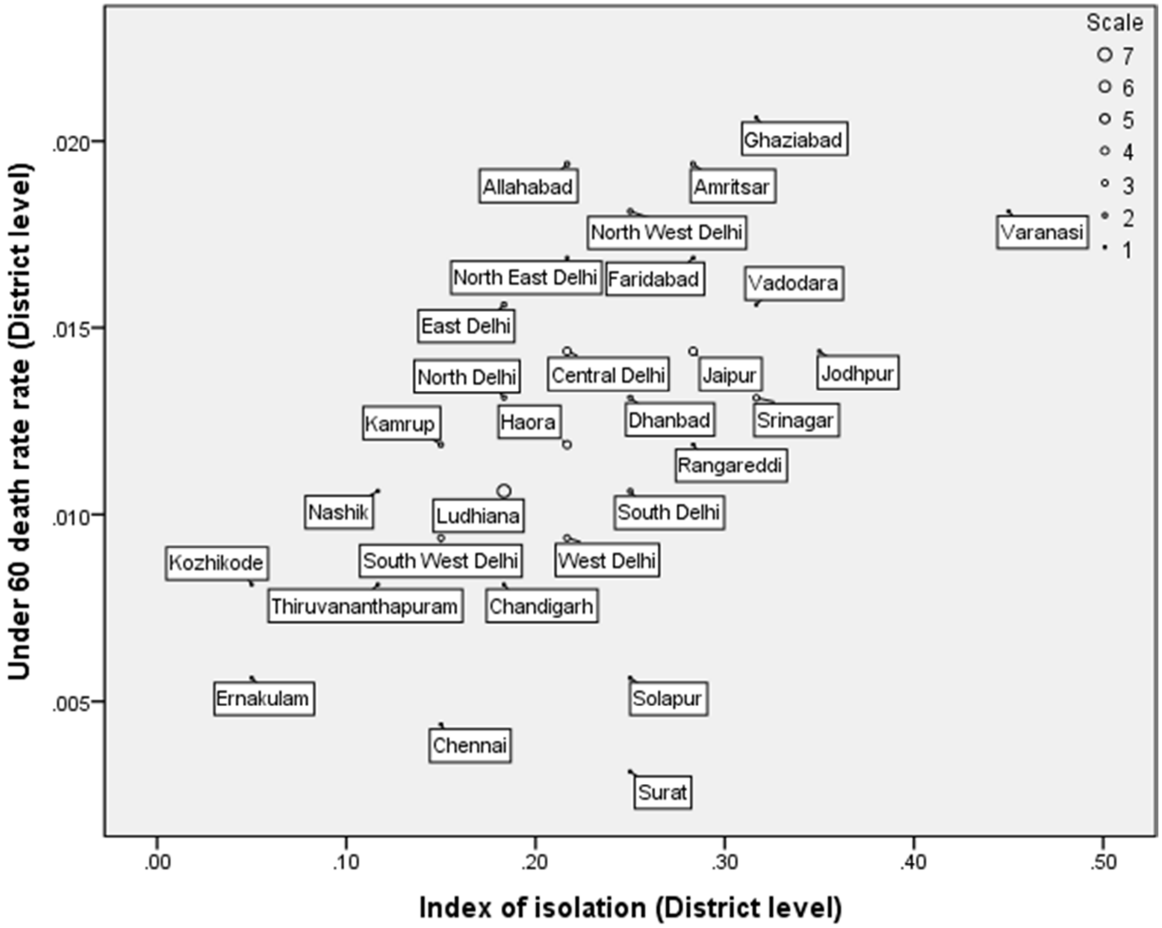

For peer review only - http://bmjopen.bmj.com/site/about/guidelines.xhtml

Figure 2: Predicted probabilities of under 60 mortality from DLHS-3 by Index of socioeconomic isolation and household literacy: probabilities estimated from Model 3, Table 2.

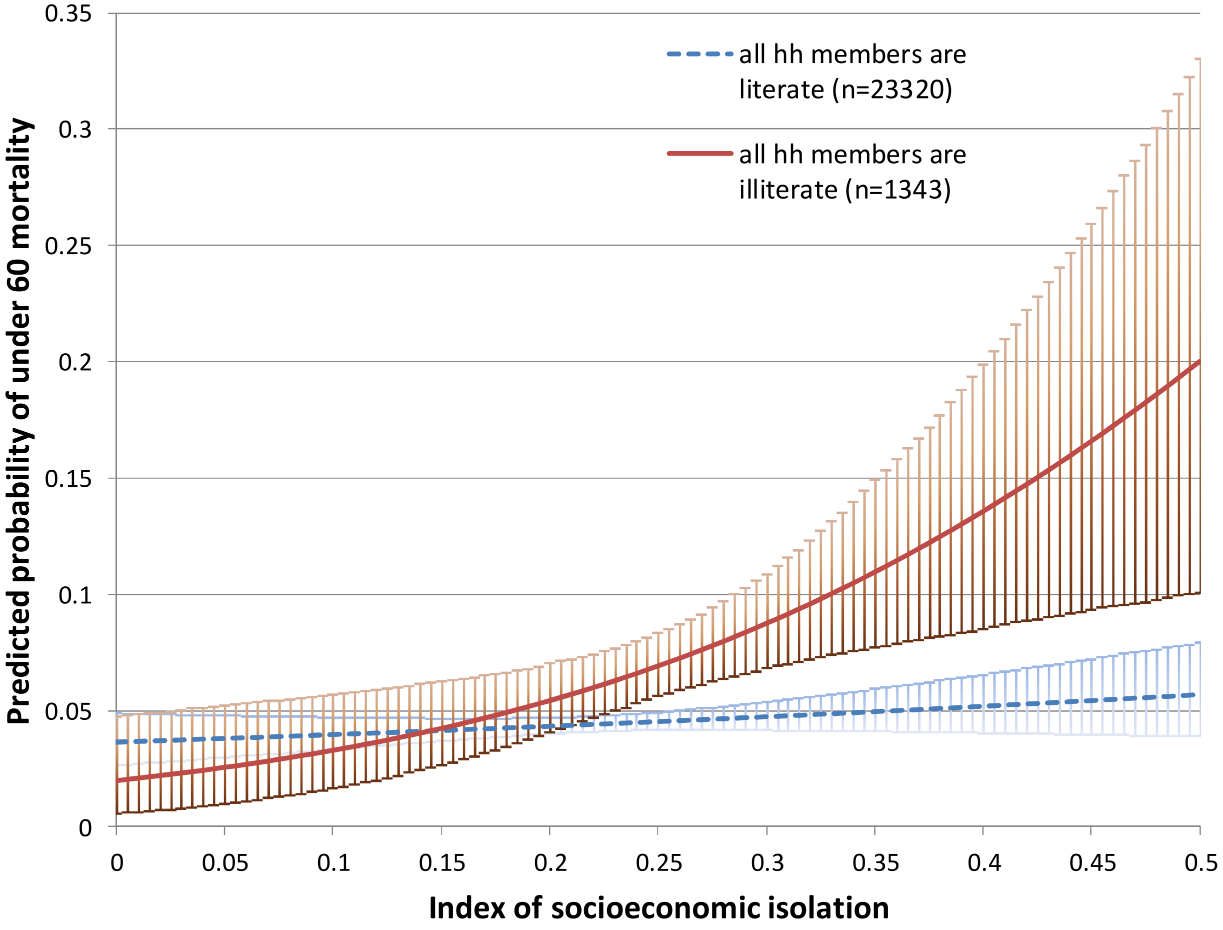

For peer review only - http://bmjopen.bmj.com/site/about/guidelines.xhtml

Appendix 1: Examining the isolation index of socioeconomic segregation in Delhi Districts

Previous analyses on the spatial distribution of slums in Delhi has shown that the South Delhi District area has the largest concentrations of slums, while the central business district area (or New Delhi District) has the smallest concentration of slums (Ishtiyaq and Kumar 2011). South Delhi is where there exists the largest concentration of squatter settlements in Delhi, which is partly because of the proximity to high income residential neighbourhoods and commercial centres. New Delhi is the central business district of the metropolis with most of the central administrative offices. With an organized and controlled developed zone as well as lack of unorganized or unusual open spaces, this zone does not provide favourable place for squatter settlements. Other studies have also commented on the low concentration of slum households in South West Delhi areas (JNRUM 2006).

Figure 2 examines the scatterplot of the illiteracy rate and index of isolation for the Delhi districts estimated from the DLHS-3 data. Although there appeared to be large differences between Delhi districts in the illiteracy rate, this is largely due to the scale of the y-axis. The maximum difference in illiteracy rates between Delhi districts was around 4%, and considerably smaller than the differences between other Indian districts shown in Figure 1 of the main manuscript. South Delhi was the district where the poor are most segregated, and New Delhi and South West Delhi were the districts where the poor are least segregated. This pattern is thus consistent with previous studies on the spatial distribution of slum populations in Delhi (Ishtiyaq and Kumar 2011, JNRUM 2006).

Figure 2: Scatterplot of District Level Illiteracy Rate and Index of Isolation in 9 Delhi Districts- DLHS-3 (2008)

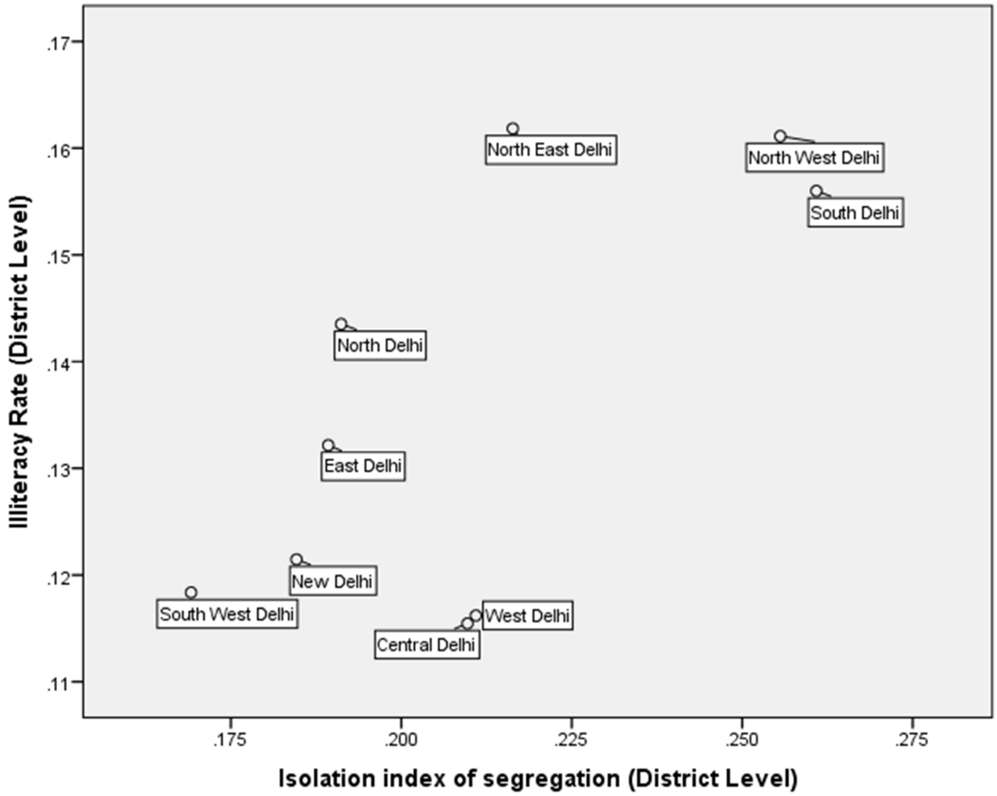

For peer review only - http://bmjopen.bmj.com/site/about/guidelines.xhtml

References:

Ishtiyaq, M. and S. Kumar, 2011. Typology of Informal Settlements and Distribution of Slums in the NCT, Delhi. J. Contemporary India Studies Space Soc. Hiroshima Univ., 1: 37-46 https://www.idosi.org/mejsr/mejsr7(6)11/33.pdf

JNRUM 2006. City development plan Delhi. Department of urban development. Government of IOndia. IL & FS Ecosmart Limited, New Delhi, India. http://ud.delhigovt.nic.in/CDPFull27.03.2007.pdf

For peer review only

BMJ Open

Is socioeconomic segregation of the poor is associated with higher premature mortality under the age of 60? A cross-sectional analysis of survey data in major Indian cities

Journal:	*BMJ Open*
Manuscript ID	bmjopen-2017-018885.R1
Article Type:	Research
Date Submitted by the Author:	10-Nov-2017
Complete List of Authors:	Chandola, Tarani; University of Manchester, The Cathie Mash Institute and Department of Social Statistics<br>Mikkilineni, Sitamma; IFHE University, IBS<br>Chandran S, Anil; University of Kerala<br>Bandyopadhyay, Souvik; Indian Institute of Public Health<br>Zhang, Nan; University of Manchester, CMIST<br>Bassanesi, Sergio; UFRGS
**Primary Subject Heading**:	Global health
Secondary Subject Heading:	Public health
Keywords:	EPIDEMIOLOGY, SOCIAL MEDICINE, PUBLIC HEALTH

SCHOLARONE™ Manuscripts

Is socioeconomic segregation of the poor is associated with higher premature mortality under the age of 60? A cross-sectional analysis of survey data in major Indian cities

Tarani Chandola, tarani.chandola@manchester.ac.uk , Cathie Marsh Institute and Department of Social Statistics, Manchester, University of Manchester
Sitamma Mikkilineni, sitamma.m@gmail.com , IBS Business School, Hyderabad, IFHE University
Anil Chandran S, anilchandrans@gmail.com , Department of Demography and Population Studies, Thiruvananthapuram, University of Kerala
Souvik Kumar Bandyopadhyay, souvik.kumar@iiph-hydedu.in , Indian Institute of Public Health, Hyderabad, Public Health Foundation of India
Nan Zhang, nan.zhang-2@manchester.ac.uk , Cathie Marsh Institute and Department of Social Statistics, Manchester University of Manchester
Sergio Luiz Bassanesi, sergio.bassanesi@ufrgs.br , Departamento de Medicina Social Porto Alegre, Universidade Federal do Rio Grande do Sul

Corresponding author:
Tarani Chandola <tarani.chandola@manchester.ac.uk>
Cathie Marsh Institute and Department of Social Statistics,
HBS Building,
University of Manchester
Manchester M13 9PL
Tel: +44 161 306 6903
Fax: +44 161 275 4722

Keywords: urban health, social segregation, socioeconomic factors, premature mortality
Word count: 3501
Reference count: 27

For peer review only

ABSTRACT

Objectives: Although urbanisation is generally associated with poverty reduction in developing counties, it also results in increased socioeconomic segregation of the poor. Cities with higher levels of socioeconomic segregation tend to have higher mortality rates, although the evidence is based on ecological associations. The paper examines whether socioeconomic segregation of the poor is associated with higher under-60 years ("premature") mortality risk in Indian cities and whether this association is confounded by contextual and compositional sociodemographic and socioeconomic factors.

Setting and participants: A population representative sample of over one million from 39,427 households living in 1,876 urban wards within 59 Indian districts (cities) from the third (2008) District Level Household Survey.

Primary outcome and other measures: The outcome was any death under the age of 60 reported by households in the preceding 4 years of the DLHS-3 survey. Socioeconomic segregation, estimated at the district (city) level, was measured using an isolation index of the poor and the index of dissimilarity.

Results: Poor households living in cities where the poor were more isolated had higher probabilities of premature mortality than poor households living in cities where the poor were less isolated. In contrast, it did not matter whether rich households lived in more or less socioeconomically segregated cities. A one standard deviation increase in the isolation index was associated with an absolute increase of 1.1% in the probability of premature mortality for the poorest households.

Conclusion: Increasing segregation of the poor may result in higher premature mortality. As developing countries become increasingly urbanised, there is a risk that this may lead to increased segregation of the poor as well as increased premature mortality.

For peer review only

Strengths and limitations

- The study examines how residential socioeconomic segregation is related to premature mortality.
- This is an important topic because urbanisation in developing countries is accompanied by an increasing spatial concentration of poverty in cities.
- For poor households, the increased risk of premature mortality is associated with urban socioeconomic segregation remains even after taking into account household and city level socioeconomic and sociodemographic confounders.
- This is a cross-sectional study so we were unable to account for unobserved factors that could cause the association between socioeconomic segregation and higher premature mortality.
- We have not estimated any lagged effects of socioeconomic segregation on premature mortality

For peer review only

For peer review only - http://bmjopen.bmj.com/site/about/guidelines.xhtml

INTRODUCTION

Urbanisation in developing countries is accompanied by increasing spatial concentration of poverty in cities. Although urbanisation can play a positive role in overall poverty reduction, an increase in the socioeconomic segregation of poor people living in cities may have adverse outcomes in terms of their chances for good health and life opportunities. While levels of urbanisation in India are relatively low at around 31 percent in 2011, the process of urbanisation is among the fastest in the world. As a result of urbanisation, India contains the highest number of urban slum dwellers, accounting for 17 per cent of the world's slum dwellers [1].

The association between poverty and ill health is well documented. The risk of premature mortality and disease as a result of living in poor areas such as slums with inadequate housing, lack of access to safe water and adequate sanitation, is well known. Furthermore, there are now a multitude of studies showing associations between area level poverty rates and deprivation and poor health/mortality, even after taking into account individual and household level socioeconomic circumstances [2-4]. This suggests an area or contextual effect on health associated with living in a poor area, which is over and above the compositional effect of the number of poor people in an area. What is perhaps less well known is the association between socioeconomic segregation and health. Studies have shown that there are ecological correlations between socioeconomic segregation by income and mortality rates [5-8]. These studies have shown that cities where there is greater socioeconomic segregation of the poor tend to have higher mortality rates. Furthermore, it is also likely that poor people living in more socioeconomically segregated cities have poorer health than other poor people who are living in less segregated cities. The concept of a 'triple health jeopardy' [5] of being poor, living in a poor district that is socioeconomically segregated from the rest of the city has been suggested. But there has been little empirical evidence linking individual mortality records to household poverty and area level segregation.

There may be a number of mechanisms underlying the relationship between socioeconomic segregation and mortality. Poor nutrition, sub-standard housing conditions and overcrowding increase susceptibility and exposure to infectious diseases [9-12]. Poor people living in socioeconomically segregated cities may have less access to good quality public services such as roads and transport infrastructure and restricted access to primary care services [5, 7, 13] compared to poor people living in less segregated cities. Poor neighbourhoods are more likely to be exposed to environmental pollutants [14-17], and their inhabitants may consequently have higher risks of respiratory and cardiovascular diseases and cancers [18, 19].

However, there are few, if any analyses that take into account potential confounders of the ecological correlation between cities with higher levels of socioeconomic segregation and mortality rates. An

For peer review only - http://bmjopen.bmj.com/site/about/guidelines.xhtml

For peer review only

apparent contextual effect of living in socioeconomically segregated cities on mortality may actually be a reflection of individual and household level associations between socioeconomic circumstances and health. Furthermore, the association between socioeconomic segregation and mortality may also be confounded by other contextual level factors such as poverty rates in the city. As people from poor households tend to live in poorer neighbourhoods and are also more likely to live in poorer cities, any association between socioeconomic segregation and health needs to take account of such potential confounding from socioeconomic factors at the compositional (household or individual) as well as contextual (city) levels.

The main research question of this paper is to investigate whether being poor, living in a poor area that is socioeconomically isolated from the rest of the city is associated with greater mortality risk compared to poor people living in less socioeconomically segregated areas in India.

METHODS

Datasets

Data from the third District Level Household Survey [20] were analysed, with the survey conducted between 2007 to 2008. The District Level Household Survey was designed to provide information on reproductive and child health in all the districts of India. The data are made available on request through the website http://rchiips.org/obtainingdata.html. There are no additional unpublished data used in this manuscript. The steps involved in the sampling of households were:

(i) the selection of primary sampling units (PSUs) within census wards;

(ii) the selection of the households from each of the selected village/urban PSUs through a random selection of households within a census enumeration block (which we have used to define as a neighbourhood in this study).

The overall household response rate, the number of households interviewed per targeted 100 households, was 94 percent. The DLHS-3 PSUs were made available for secondary data analyses, allowing the estimating of neighbourhood socio-economic segregation indices.

For this study, the analytical sample was restricted to households living within urban wards of the largest cities with a population of more than 1 million. This resulted in an analytical sample of 39,446 households living in 1,876 urban wards within 59 cities. This was to ensure comparability of the urban units within the analysis. Mega cities such as Kolkata, Mumbai and Delhi were disaggregated into their constituent administrative districts.

Outcome

Premature mortality under the age 60 was reported for each household participating in the DLHS-3 in the preceding four years (from 2004-2008). This was recoded into a binary variable (any premature death in the

For peer review only - http://bmjopen.bmj.com/site/about/guidelines.xhtml

For peer review only

household vs. no premature death) for the probit regression analysis. The under-60 death rate was calculated at the district level by dividing the total premature mortality in a district by the number of respondents aged under-60 in the DLHS-3 sample.

Measures of socioeconomic segregation

There are a number of measures for segregation, measuring different dimensions of the concept. In this study, two measures of segregation were used- the isolation index of segregation and the index of dissimilarity.

The Isolation Index of Segregation

The study used estimates of neighbourhood socio-economic composition from DLHS-3 districts and primary sample units (PSUs). PSUs correspond to around 300 households from a census enumeration block and we used them to represent urban "neighbourhoods". Poverty was measured through the proxy indicator of illiteracy as direct measures of poverty were not available in the data. The index of isolation was used to measure the socioeconomic segregation of the poor. The isolation index (Ind_isolation_j) measures the extent to which poor members of a district are exposed only to one another and is computed as the weighted average of the proportion of poor people in each neighbourhood [21].

$$\text{Ind\_isolation}_j = \sum_{i=1}^{j} \frac{\text{illiterate}_{ij}}{\text{illiterate}_j} \times \frac{\text{illiterate}_{ij}}{\text{population}_{ij}}$$

where

illiterate_{ij} is the number of illiterate adults living in neighbourhood_i within district_j

Illiterate_j is the number of illiterate adults living in district_j

Population_{ij} is the adult population living in neighbourhood_i within district_j

As the isolation index is a weighted poverty rate of the district, it is strongly influenced by the (unweighted) poverty rate in a district. It can be adjusted to control for the effect of population composition in the city by the following way:

$\text{Ind\_isolation\_adj}_j = (\text{Ind\_isolation}_j — P)/(1— P)$

Where P is the proportion of poor people in the city.

This adjusted isolation index has also been suggested as a potential measure of segregation in its own right. Stearns and Logan (1986) go so far as to suggest that it represents an independent dimension of segregation[22].

Index of dissimilarity

For peer review only - http://bmjopen.bmj.com/site/about/guidelines.xhtml

Another measure of segregation in this study is the index of dissimilarity which focuses on residential evenness [23]. It measures the evenness with which poor and rich groups are distributed across the neighbourhoods that make up a city.

The index indicates the proportion of the poor population that would have to move neighbourhoods in order to achieve an even distribution within the city.

$$\text{Ind\_dissimilarity}_j = 0.5 \left| \sum_{i=1}^{j} \frac{\text{illiterate}_{ij}}{\text{illiterate}_j} - \frac{\text{not illiterate}_{ij}}{\text{not illiterate}_j} \right|$$

For all the segregation measures, the DLHS-3 survey weights were used to derive the district and neighbourhood level estimates of the illiterate, not illiterate and total populations. As the DLHS-3 does not contain information on all neighbourhoods within a district, the estimated indices may not accurately reflect the true distribution of the district level segregation indices. However, the use of the DLHS-3 survey data does allow for an estimate of segregation using data from smaller geographical areas (through the random sample of households within a primary sampling unit), rather than estimating the Index using data from larger census area levels such as the ward level, which often refers to a very large area and population (around 25,000-75,000) for the largest Indian cities.

Covariates

As there are no direct measures of income or poverty in the DLHS data, poverty was measured through the use of a proxy indicator namely illiteracy. Another reason for using illiteracy to measure poverty is the difference in the meanings and concepts of standard poverty measures (such as indicators of wealth and status) across Indian cities. The illiteracy rates were calculated by dividing the total number of illiterate adults in a DLHS-3 household or district by the number of adults in that household or district. Other potential sociodemographic and socioeconomic confounders included the number of household members, household wealth (constructed by the DLHS-3 survey team from a factor analysis of consumption measures and housing quality indicators), and religion of head of household, whether the head of household belonged to a scheduled caste (SC) or scheduled tribe (ST), and whether the household accessed government or private health care when someone was ill. City population (per 100,000 inhabitants) and proportion of city population that belonged to scheduled caste or schedule tribe groups were derived from the weighted DLHS-3 district level estimates.

Analysis

The ecological (city level) correlation between the district level under-60 mortality rate, the poverty rate and the three socioeconomic segregation measures were examined. Multilevel probit regression (with the

For peer review only - http://bmjopen.bmj.com/site/about/guidelines.xhtml

outcome of under-60 death within a household) was used to estimate the effect of the (district level) the three measures of socioeconomic segregation, after taking into account socioeconomic, sociodemographic factors at the individual and household levels, as well as city level socioeconomic factors (the poverty rate, the population and proportion of population that are SCST). Multilevel analysis is particularly appropriate as it takes into account the sample design of the DLHS-3 which results in clustered samples at the state, district and neighbourhood (PSU) levels- each of these levels were specified with a random intercept in the multilevel models. It is important to take this clustering into account in the analysis as the main exposure variable is at the district level, rather than at the household level. The multilevel regression coefficients were estimated using the PQL2 estimation procedures within MLwin.

The question of whether being poor, living in a poor area that is socioeconomically segregated from the rest of the city is associated with greater mortality risk, was examined through an interaction between the household illiteracy rate ("being poor") with the measures of socioeconomic segregation.

RESULTS

The scatterplot of the district level associations between premature death rates and the unadjusted Index of Isolation (Figure 1) shows that there was a moderate ecological correlation (R=0.52). Districts (cities) where the poor were more isolated were also those with higher premature death rates. Only a few of the 59 districts are displayed in the scatterplot to aid ease of interpretation. Some of the districts such as North West Delhi and West Delhi had the same levels of the isolation index, but differed in terms of premature death rates by more than 5%. The ecological correlations (for all the 59 districts) between all the district level measures are shown in Table 1. The correlation between the (unadjusted) index of isolation and illiteracy rate was very strong (r=0.90) which meant that using both in a regression analysis would be likely to cause multicollinearity problems. In contrast, there was a weak correlation between the adjusted index of isolation and the illiteracy rate, and a strong correlation between the adjusted index of isolation and the dissimilarity index.

For peer review only - http://bmjopen.bmj.com/site/about/guidelines.xhtml

Table 1: Ecological correlation between district level under 60 death rate, illiteracy rate and measures of socioeconomic segregation

	under age 60 death rate	Illiteracy rate	Index of isolation (unadjusted)	Index of isolation (adjusted)	Index of dissimilarity
under age 60 death rate	1				
Illiteracy/Poverty rate	0.50	1			
Index of isolation (unadjusted)	0.52	0.90	1		
Index of isolation (adjusted)	0.30	0.29	0.68	1	
Index of dissimilarity	-0.15	-0.34	0.04	0.67	1

Table 2 displays the distribution of the key independent variables in the analysis by any premature death in the household. 5.3% of all households in the analytical sample reported the death of a household member aged under 60 in the four years period preceding the survey. Households where there was a premature death in the preceding 4 years were living in districts with higher Index of isolation scores (unadjusted and adjusted indices), lower dissimilarity index scores, higher illiteracy rates at the district and household levels, lower wealth scores, more household members, were more likely to be Muslim and Scheduled Caste/Scheduled Tribe households and also were more likely to use government clinics when ill.

For peer review only - http://bmjopen.bmj.com/site/about/guidelines.xhtml

Table 2 Distribution (Mean and Percentages) of key independent variables in the analysis by premature death (under 60) in the household: DLHS-3

	No death under 60 in the household	≥1 death under 60 in the household
n	*37346*	*2100*
Isolation Index (District level)	0.22 (0.06)	0.23 (0.06)
Isolation Index adjusted (District level)	0.08(0.03)	0.09 (0.04)
Dissimilarity Index (District level)	0.33 (0.07)	0.32 (0.07)
Illiteracy rate (District level)	0.15 (0.05)	0.16 (0.05)
Illiteracy rate (Household level)	0.16 (0.24)	0.23 (0.29)
Mean wealth index score	1.33 (0.93)	1.05 (0.92)
Mean number of household members	4.92 (2.37)	5.25 (2.78)
% of households that are Muslim	16.4%	19.3%
% of households that are SC/ST	18.4%	24.1%
% of households using government clinics when ill	38.4%	41.9%

SC: Scheduled Caste; ST: Scheduled Tribe; DLHS: District Level Household Survey.

Table 3 displays the results (estimated probits and 95% confidence intervals) of the multilevel probit regression models with any premature death in the household as the dependent variable. In Model 1, the model with all the household level sociodemographic and socioeconomic factors was fitted, taking into account the clustering in premature mortality at the district, primary sampling unit (PSU) and household levels. This probit model allows for extra-binomial variance at the lowest (household) level as the variance of the distribution of household mortality was greater than expected under the binomial assumption. There appeared to be some clustering in premature mortality at the PSU level, but a negligible amount of clustering at the district level. We additionally examined whether adding in Indian states as an additional level was needed in the multilevel analyses, but found little evidence of clustering in premature mortality at the state level.

Being a member of an illiterate or poorer household with more household members, and living in the Northern region were associated with increased probabilities of premature mortality. Furthermore, living in a city with a higher proportion of households belonging to scheduled castes and scheduled tribes was associated with higher probabilities of premature mortality, although the population size of cities did not predict mortality. While the main effect of the district level poverty rate on premature mortality was positive and significant (analysis not shown), the interaction term between district level poverty rate and the household level poverty rate was non-significant. This suggests that while there are additive but not multiplicative effects of living in a poor (illiterate) household and poor (illiterate) city on the risks of premature mortality.

For peer review only - http://bmjopen.bmj.com/site/about/guidelines.xhtml

For peer review only

Model 2 added in the unadjusted Isolation Index (in standard deviation unit differences from the mean) and the interaction between the (unadjusted) Isolation index and the household illiteracy rate as independent variables. A one SD increase in the isolation index was associated with a non-significant increase of 0.02 in the probit of premature mortality for the richest (non-illiterate) households. Whereas for the poorest (all illiterate) households, an increase in 1 SD of the (unadjusted) isolation index was associated with a significant increase of 0.15 in the probit of premature mortality (0.02+0.13). As the unadjusted isolation index and the district level illiteracy rate are strongly correlated, these probit estimates may suffer from collinearity issues. Hence we used the adjusted isolation index in Model 3 to avoid potential collinearity issues with the district level illiteracy rate. Here again, there was some evidence of an interaction between the (adjusted) isolation index and household level illiteracy rate- poor households (where all the members were illiterate) living in more isolated cities (a one SD increase in the adjusted isolation index) had significantly higher probits of premature mortality (0.10) compared to poor households living in cities where the poor were less isolated. The estimated probabilities from Model 3 are graphed in Figure 2. Although richer (completely literate) households have lower probabilities of premature mortality than poorer (completely illiterate) households, the poorest households which live in cities where the poor are less isolated have significantly lower probabilities of premature mortality than the poorest households living in cities where the poor are more isolated. The estimated probability of premature mortality for the poorest households living in cities where the adjusted isolation index was 1 SD below the average for all the Indian cities was 5.5%. At average levels of of the adjusted isolation index, the probability of premature mortality for the poorest households was 6.6%- an absolute difference of 1.1%. In contrast, for rich households, there was hardly any difference in the predicted probability of premature mortality in terms of living in city where the poor were more or less isolated. A very similar interaction effect was found in Model 5 for the combination of poor households living in cities with a greater dissimilarity index, but the interaction term was borderline significant.

For peer review only

For peer review only - http://bmjopen.bmj.com/site/about/guidelines.xhtml

Table 3 Probits (and 95% confidence intervals) from Multilevel Probit Regression Models of premature mortality: DLHS-3

Fixed Part	**Model 1**	**Model 2**	**Model 3**	**Model 4**
Intercept	**-1.60 (-1.67, -1.53)**	**-1.59 (-1.66, -1.52)**	**-1.59 (-1.66, -1.52)**	**-1.59 (-1.66, -1.52)**
Household Illiteracy rate (ref: none illiterate)				
Some members are illiterate	**0.10 (0.04, 0.15)**	**0.10 (0.04, 0.15)**	**0.09 (0.04, 0.15)**	**0.10 (0.05, 0.15)**
All members are illiterate	**0.17 (0.05, 0.29)**	**0.16 (0.04, 0.28)**	**0.19 (0.08, 0.30)**	**0.22 (0.11, 0.32)**
Wealth tertiles (ref: poorest tertile)				
Middle wealth tertile	**-0.11 (-0.16, -0.06)**	**-0.11 (-0.16, -0.06)**	**-0.11 (-0.16, -0.06)**	**-0.11 (-0.16, -0.06)**
Richest tertile	**-0.25 (-0.32, -0.19)**	**-0.25 (-0.32, -0.19)**	**-0.25 (-0.31, -0.19)**	**-0.25 (-0.31, -0.19)**
Region (ref: North)				
East	**-0.15 (-0.23, -0.06)**	**-0.15 (-0.24, -0.07)**	**-0.15 (-0.23, -0.06)**	**-0.15 (-0.24, -0.07)**
West	**-0.11 (-0.19, -0.03)**	**-0.11 (-0.19, -0.03)**	**-0.11 (-0.19, -0.03)**	**-0.11 (-0.19, -0.03)**
South	**-0.23 (-0.31, -0.15)**	**-0.23 (-0.31, -0.14)**	**-0.23 (-0.31, -0.14)**	**-0.23 (-0.31, -0.15)**
Religion (ref: Hindu)				
Muslim	0.02 (-0.04, 0.08)	0.02 (-0.04, 0.09)	0.02 (-0.04, 0.09)	0.02 (-0.04, 0.09)
Other religion	0.03 (-0.06, 0.12)	0.03 (-0.06, 0.12)	0.03 (-0.06, 0.12)	0.03 (-0.06, 0.12)
Caste (ref: other caste household)				
SCST caste hh	**0.11 (0.05, 0.17)**	**0.11 (0.05, 0.17)**	**0.11 (0.05, 0.17)**	**0.11 (0.05, 0.17)**
Other backward caste hh	0.05 (-0.01, 0.10)	0.04 (-0.01, 0.10)	0.04 (-0.01, 0.10)	0.05 (-0.01, 0.10)
Missing/DK caste hh	-0.05 (-0.22, 0.13)	-0.05 (-0.23, 0.13)	-0.05 (-0.23, 0.12)	-0.05 (-0.23, 0.13)
Access to private health care (ref: yes)				
Usually go to govt clinic when ill	0.01 (-0.03, 0.06)	0.01 (-0.03, 0.06)	0.01 (-0.03, 0.06)	0.01 (-0.03, 0.06)
Number of people in the household	**0.01 (0.003, 0.02)**	**0.01 (0.003, 0.02)**	**0.01 (0.003, 0.02)**	**0.01 (0.003, 0.02)**
% of pop who are SCST (District level)	**0.004 (0.0003, 0.01)**	**0.004 (0.0001, 0.01)**	**0.004 (0.0001, 0.01)**	**0.004 (0.00003, 0.008)**
Population per 100,000 (District level)	0.0001 (-0.002, 0.002)	0.0001 (-0.002, 0.002)	0.00003 (-0.002, 0.002)	0.00004 (-0.002, 0.002)
Illiteracy rate (District level)	0.63 (-0.07, 1.34)	0.24 (-0.81, 1.29)	**0.73 (0.17, 1.29)**	**0.84 (0.28, 1.41)**
Interaction of Illiteracy rate (District level) and Illiteracy rate (household)				
Illiteracy rate*Some illiterate	0.14 (-0.77, 1.06)			
Illiteracy rate*All illiterate	1.89 (-0.24, 4.01)			
Unadjusted Isolation index (District level)		0.02 (-0.04, 0.07)		
Interaction of Unadjusted Isolation Index and (District level) and Illiteracy rate (household)				
Unadjusted Isolation Index*Some illiterate		0.02 (-0.03, 0.07)		
Unadjusted Isolation Index*All illiterate		**0.13 (0.02, 0.23)**		
Adjusted Isolation index corrected (District level)			-0.004 (-0.04, 0.03)	
Interaction of Adjusted Isolation Index adjusted and (District level) and Illiteracy rate (household)				
Adjusted Isolation Index*Some illiterate			0.03 (-0.01, 0.07)	
Adjusted Isolation Index*All illiterate			**0.10 (0.01, 0.19)**	
Dissimilarity index (District level)				-0.01 (-0.04, 0.03)
Interaction of Dissimilarity Index and (District level) and Illiteracy rate (household)				
Dissimilarity Index*Some illiterate				0.03 (-0.02, 0.07)
Dissimilarity Index*All illiterate				0.11 (-0.0005, 0.22)
Random Part				
District variance (n=59)	0.013 (0.014)	0.013 (0.014)	0.013 (0.014)	0.013 (0.014)
PSU variance (n=1876)	0.038 (0.017)	0.037 (0.017)	0.036 (0.017)	0.036 (0.017)
Household extra binomial variance (n=39427)	0.934 (0.007)	0.934 (0.007)	0.935 (0.007)	0.936 (0.007)

DLHS: District Level Household Survey; SC: scheduled Caste; ST: Scheduled Tribe.

Statistically significant ($p<0.05$) probit coefficients are shown in **bold**

For peer review only - http://bmjopen.bmj.com/site/about/guidelines.xhtml

DISCUSSION

This study found that urban Indian districts where the poor were segregated (i.e. isolated or unevenly distributed) had higher premature mortality rates than districts where the poor were less segregated. Poor households living in cities where the poor were less isolated (cities which were 1 standard deviation lower than the average adjusted isolation index of Indian cities) had around 1.1% lower probabilities of premature mortality than poor households living in cities where the poor were more isolated. This association appeared to remain even after taking into account a range of compositional (household level) and contextual (district level) socio-demographic and socio-economic factors that could confound this association. There thus appears to be some evidence of a 'triple health jeopardy' [5] of being poor, living in a poor district that is socioeconomically isolated from the rest of the city.

In India, the top causes of mortality include non-communicable causes like cardiovascular diseases, cancer and respiratory diseases, as well as infectious diseases like tuberculosis, malaria and diarhheoa, and injuries (suicide and accidents) [24]. Poor people living in cities where the poor are less isolated may be less exposed to environmental pollutants that cause respiratory diseases and cancers and they may benefit from the shared living spaces with richer people. Conversely, poor people living in cities where the poor are more isolated may be far from good transport and green spaces, and have little access to water, sewage, sanitation and good quality health services in the neighbourhoods where they live. Even though they live in the same city, because the neighbourhoods they live in are very isolated from rich people, so they cannot access the same services as the rich people do. This association between socioeconomic segregation and premature mortality is independent of how poor the city is as a whole, and thus the association appears to be related to the residential distribution of poor people in a city, rather than how rich or poor the city is. Socioeconomic segregation may enable the affluent access to the greatest resources and reinforce the social exclusion of the disadvantaged [21, 25, 26]. Income segregation may reinforce social inequality [27] through segregating the poor away from resources such as employment, transport, education, health care and other services, a process which could discourage social mobility. It may thus capture dimensions of social inequality distinct from poverty or deprivation rates that have typically been used to measure social inequality at an area level.

There are a number of limitations to this study. This is a cross-sectional study showing associations between premature mortality and a single measure of socioeconomic segregation. We were unable to infer causality from observational evidence as we have not observed whether increasing socioeconomic segregation results in higher premature mortality. While we have adjusted for a range of potential socio-economic confounders at the district and household levels, there may be other unobserved factors, including selection effects, that cause the association of socioeconomic segregation with premature mortality. In addition, we have not estimated any lagged effects of socioeconomic segregation on

For peer review only - http://bmjopen.bmj.com/site/about/guidelines.xhtml

premature mortality. If the association is causal, we do not know what duration of exposure to a socioeconomically segregated neighbourhood is needed to generate higher premature mortality risks. The measure of socioeconomic segregation was estimated from survey data and so may not be accurately estimated. Appendix 1 examines the association of the index of isolation with the district level illiteracy rates for the Delhi districts, where there have been some studies on the spatial distribution of slum housing. The analysis confirms the expected ranking of Delhi districts by the index of isolation, suggesting that the isolation index estimated by the District Level Household Survey may be valid. Other dimensions of socioeconomic segregation could not be measured due to data limitations. In particular, we have not been able to measure the spatial dimension of socio-economic segregation which may be particularly relevant in relation to discussions of social inequalities. The outcome measure, under 60 mortality, was not age-specific, resulting in different causes of deaths being grouped together in a crude metric. This was because of the small numbers of deaths at the household level. If we had attempted more age-specific mortality analyses, this would have resulted in unstable models with zero cell counts.

As urbanisation increases in India, there is a risk of increasing socioeconomic segregation of the poor and associated health risks. Indian cities already contain the largest concentration of the world's population living in slums. With increasing urbanisation and associated economic pressures, the poor living within Indian cities are at risk of increasing isolation such as through slum resettlement programs far from employment, transport and public services networks. The results from this study cautions against such policies that increase the socioeconomic segregation of the poor, as this may lead to increased levels of premature mortality among the most vulnerable groups in society.

Contributors: TC, SKB and SM contributed to the conception and design of the study. ACS, SLB and NZ contributed to the analysis and interpretation of the data. TC drafted the initial manuscript and SLB, SM, SKB, ACS and NZ contributed to revisions of the manuscript. All authors gave approval for the final version and agree to be accountable for all aspects of the study.

Funding This work was supported by the Economic and Social Research Council (ESRC): RES-238-25-0015 and ES/J019119/1

Competing interests: None declared

For peer review only

REFERENCES

1 Un-habitat. *State of the world's cities 2010/2011: bridging the urban divide*. London: Earthscan 2010.
2 Van Ham M, Manley D, Bailey N*, et al. Understanding neighbourhood dynamics: New insights for neighbourhood effects research*. London: Springer 2012.
3 Pickett KE, Pearl M. Multilevel analyses of neighbourhood socioeconomic context and health outcomes: a critical review. *Journal of Epidemiology and Community Health* 2001;**55**:111-22.
4 Kawachi I, Berkman LF. *Neighborhoods and health*: Oxford University Press 2003.
5 Ross NA, Nobrega K, Dunn J. Income segregation, income inequality and mortality in North American metropolitan areas. *GeoJournal* 2001;**53**:117-24.
6 Waitzman NJ, Smith KR. Separate but lethal: the effects of economic segregation on mortality in metropolitan America. *Milbank Quarterly* 1998;**76**:341-73.
7 Lobmayer P, Wilkinson RG. Inequality, residential segregation by income, and mortality in US cities. *Journal of Epidemiology and Community Health* 2002;**56**:183-7.
8 Szwarcwald CL, de Andrade CLT, Bastos FI. Income inequality, residential poverty clustering and infant mortality: a study in Rio de Janeiro, Brazil. *Social science & medicine* 2002;**55**:2083-92.
9 Walker SP, Wachs TD, Gardner JM*, et al.* Child development: risk factors for adverse outcomes in developing countries. *The lancet* 2007;**369**:145-57.
10 Immink MDC. The urban poor and household food security: policy and project lessons of how governments and the urban poor attempt to deal with household food insecurity, poor health and malnutrition. Concepts, evidence and case studies New York: United Nations Children's Fund (UNICEF) 1994.
11 Alirol E, Getaz L, Stoll B*, et al.* Urbanisation and infectious diseases in a globalised world. *The Lancet infectious diseases* 2011;**11**:131-41.
12 Galobardes B, Lynch JW, Smith GD. Childhood socioeconomic circumstances and cause-specific mortality in adulthood: systematic review and interpretation. *Epidemiologic reviews* 2004;**26**:7-21.
13 Hassing C, Twickler M, Brunekreef B*, et al.* Particulate air pollution, coronary heart disease and individual risk assessment: a general overview. *European Journal of Cardiovascular Prevention & Rehabilitation* 2009;**16**:10-5.
14 Anderton DL, Anderson AB, Rossi PH*, et al.* Hazardous Waste Facilities" Environmental Equity" Issues in Metropolitan Areas. *Evaluation Review* 1994;**18**:123-40.
15 Ellen IG, Mijanovich T, Dillman KN. Neighborhood effects on health: exploring the links and assessing the evidence. *Journal of Urban Affairs* 2001;**23**:391-408.
16 Cohen DA, Mason K, Bedimo A*, et al.* Neighborhood physical conditions and health. *American Journal of Public Health* 2003;**93**:467-71.
17 Ash M, Fetter TR. Who Lives on the Wrong Side of the Environmental Tracks? Evidence from the EPA's Risk ‐ Screening Environmental Indicators Model*. *Social Science Quarterly* 2004;**85**:441-62.
18 Harpham T. Urban health in developing countries: what do we know and where do we go? *Health & place* 2009;**15**:107-16.
19 Roux AVD, Merkin SS, Arnett D*, et al.* Neighborhood of residence and incidence of coronary heart disease. *New England Journal of Medicine* 2001;**345**:99-106.
20 International Institute for Population Sciences (IIPS). *District Level Household and Facility Survey (DLHS-3), 2007-08: India*. Mumbai: IIPS 2010.
21 Massey DS, Denton NA. The dimensions of residential segregation. *Social forces* 1988;**67**:281-315.
22 Stearns LB, Logan JR. Measuring trends in segregation: three dimensions, three measures. *Urban affairs quarterly* 1986;**22**:124-50.
23 James DR, Taeuber KE. Measures of segregation. *Sociological methodology* 1985;**15**:1-32.

For peer review only - http://bmjopen.bmj.com/site/about/guidelines.xhtml

24 Yadav S, Arokiasamy P. Understanding epidemiological transition in India. *Global health action* 2014;**7**:23248.
25 Massey DS. The age of extremes: Concentrated affluence and poverty in the twenty-first century. *Demography* 1996;**33**:395-412.
26 Jargowsky PA. Take the money and run: Economic segregation in US metropolitan areas. *American sociological review* 1996:984-98.
27 Dwyer RE. Poverty, prosperity, and place: the shape of class segregation in the age of extremes. *Social Problems* 2010;**57**:114-37.

For peer review only

For peer review only - http://bmjopen.bmj.com/site/about/guidelines.xhtml

Figure 1: Scatterplot of District Level Under 60 death rate and the Unadjusted Index of Isolation in 59 Indian urban districts- DLHS-3 (2008): selected districts shown

Figure 2: Predicted probabilities (and 95% confidence intervals) of under 60 mortality from DLHS-3 by the adjusted index of socioeconomic isolation and household literacy: probabilities estimated from Model 3, Table 3.

For peer review only

For peer review only - http://bmjopen.bmj.com/site/about/guidelines.xhtml

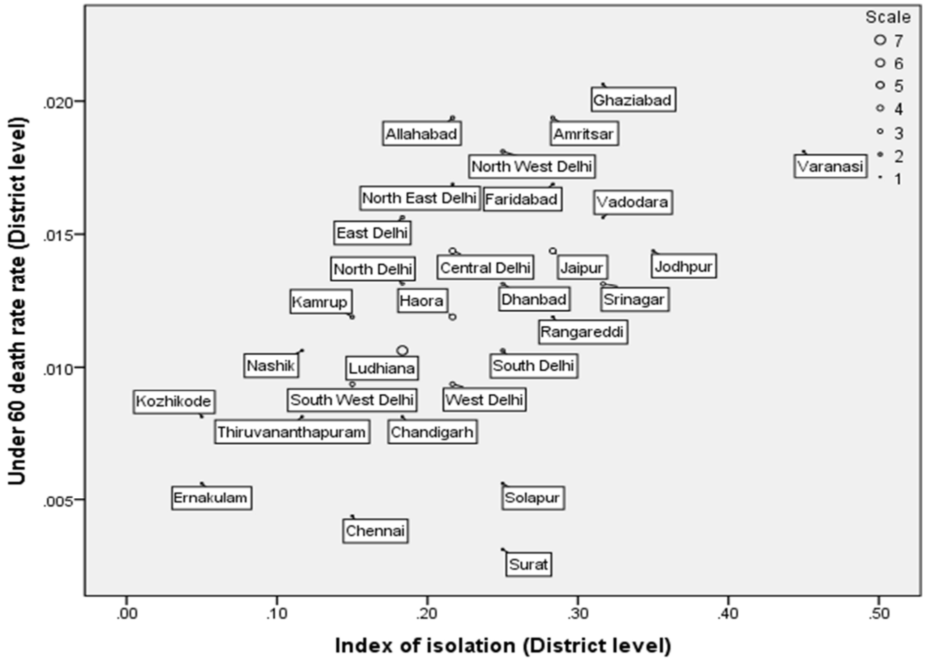

Figure 1: Scatterplot of District Level Under 60 death rate and the Unadjusted Index of Isolation in 59 Indian urban districts- DLHS-3 (2008): selected districts shown

254x190mm (300 x 300 DPI)

For peer review only - http://bmjopen.bmj.com/site/about/guidelines.xhtml

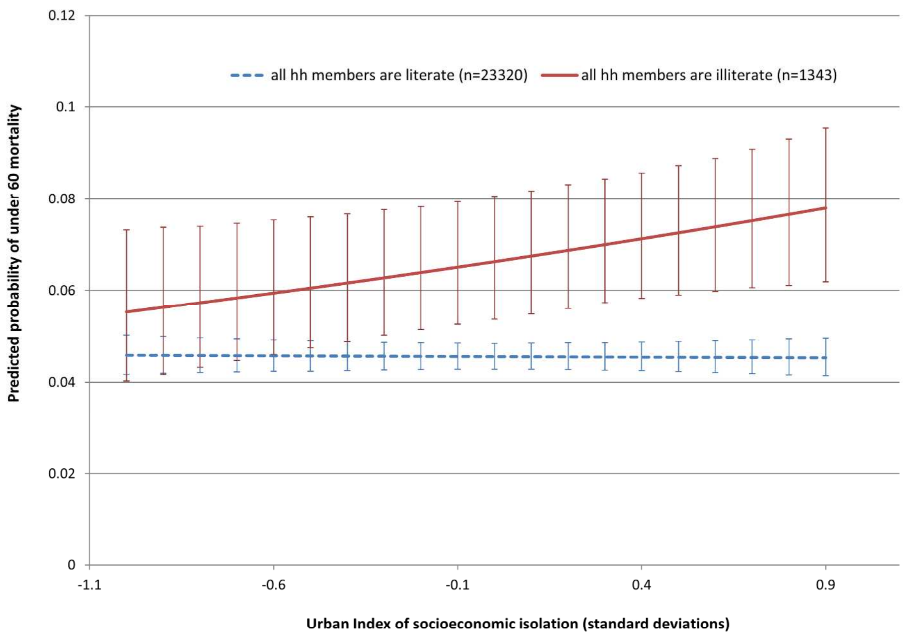

Figure 2: Predicted probabilities (and 95% confidence intervals) of under 60 mortality from DLHS-3 by the adjusted index of socioeconomic isolation and household literacy: probabilities estimated from Model 3, Table 3.

254x190mm (300 x 300 DPI)

Appendix 1: Examining the unadjusted isolation index of socioeconomic segregation in Delhi Districts

Previous analyses on the spatial distribution of slums in Delhi has shown that the South Delhi District area has the largest concentrations of slums, while the central business district area (or New Delhi District) has the smallest concentration of slums (Ishtiyaq and Kumar 2011). South Delhi is where there exists the largest concentration of squatter settlements in Delhi, which is partly because of the proximity to high income residential neighbourhoods and commercial centres. New Delhi is the central business district of the metropolis with most of the central administrative offices. With an organized and controlled developed zone as well as lack of unorganized or unusual open spaces, this zone does not provide favourable place for squatter settlements. Other studies have also commented on the low concentration of slum households in South West Delhi areas (JNRUM 2006).

Figure 2 examines the scatterplot of the illiteracy rate and the unadjusted index of isolation for the Delhi districts estimated from the DLHS-3 data. Although there appeared to be large differences between Delhi districts in the illiteracy rate, this is largely due to the scale of the y-axis. The maximum difference in illiteracy rates between Delhi districts was around 4%, and considerably smaller than the differences between other Indian districts shown in Figure 1 of the main manuscript. South Delhi was the district where the poor are most isolated, and New Delhi and South West Delhi were the districts where the poor are least isolated. This pattern is thus consistent with previous studies on the spatial distribution of slum populations in Delhi (Ishtiyaq and Kumar 2011, JNRUM 2006).

Figure 2: Scatterplot of District Level Illiteracy Rate and Index of Isolation in 9 Delhi Districts- DLHS-3 (2008)

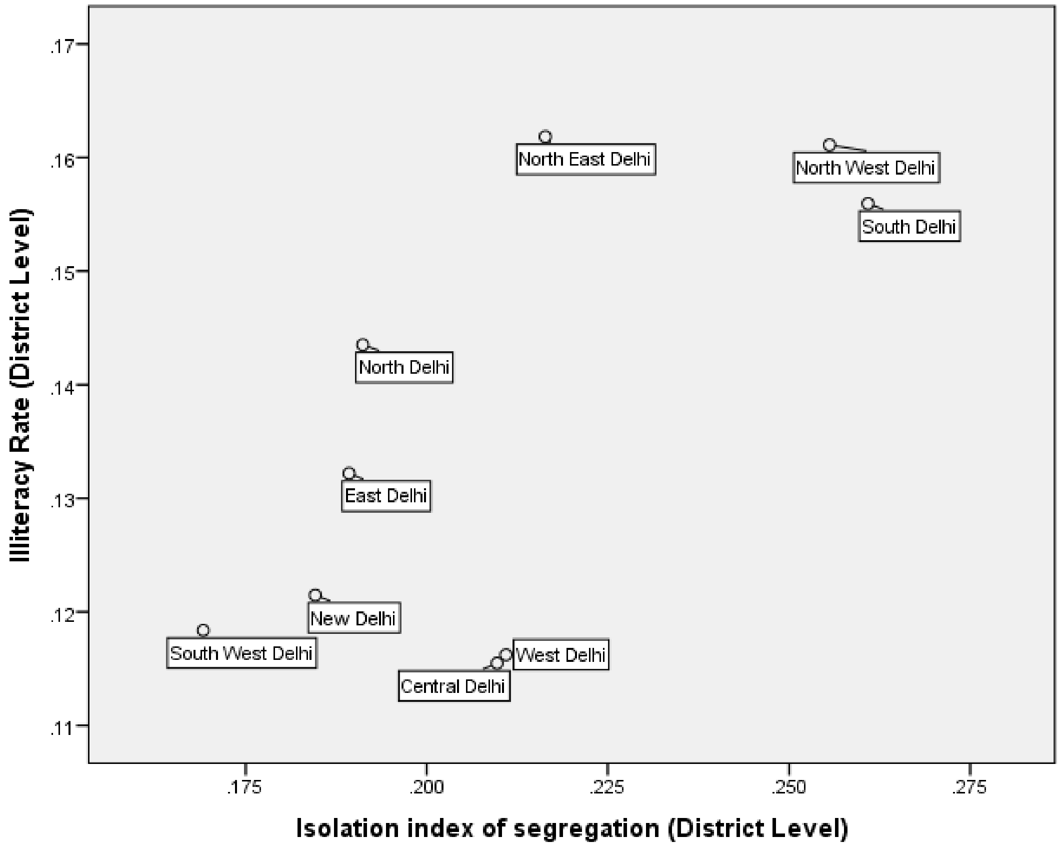

References:

Ishtiyaq, M. and S. Kumar, 2011. Typology of Informal Settlements and Distribution of Slums in the NCT, Delhi. J. Contemporary India Studies Space Soc. Hiroshima Univ., 1: 37-46 https://www.idosi.org/mejsr/mejsr7(6)11/33.pdf

JNRUM 2006. City development plan Delhi. Department of urban development. Government of IOndia. IL & FS Ecosmart Limited, New Delhi, India. http://ud.delhigovt.nic.in/CDPFull27.03.2007.pdf

For peer review only

STROBE 2007 (v4) Statement—Checklist of items that should be included in reports of *cohort studies*

Section/Topic	Item #	Recommendation	Reported on page #
**Title and abstract**	1	(*a*) Indicate the study's design with a commonly used term in the title or the abstract	1
		(*b*) Provide in the abstract an informative and balanced summary of what was done and what was found	2
**Introduction**			
Background/rationale	2	Explain the scientific background and rationale for the investigation being reported	4
Objectives	3	State specific objectives, including any prespecified hypotheses	5
**Methods**			
Study design	4	Present key elements of study design early in the paper	5
Setting	5	Describe the setting, locations, and relevant dates, including periods of recruitment, exposure, follow-up, and data collection	5
Participants	6	(*a*) Give the eligibility criteria, and the sources and methods of selection of participants. Describe methods of follow-up	5
		(*b*) For matched studies, give matching criteria and number of exposed and unexposed	NA
Variables	7	Clearly define all outcomes, exposures, predictors, potential confounders, and effect modifiers. Give diagnostic criteria, if applicable	4-5
Data sources/ measurement	8*	For each variable of interest, give sources of data and details of methods of assessment (measurement). Describe comparability of assessment methods if there is more than one group	5-7
Bias	9	Describe any efforts to address potential sources of bias	7-8
Study size	10	Explain how the study size was arrived at	5
Quantitative variables	11	Explain how quantitative variables were handled in the analyses. If applicable, describe which groupings were chosen and why	5-7
Statistical methods	12	(*a*) Describe all statistical methods, including those used to control for confounding	7-8
		(*b*) Describe any methods used to examine subgroups and interactions	7-8
		(*c*) Explain how missing data were addressed	NA
		(*d*) If applicable, explain how loss to follow-up was addressed	NA
		(*e*) Describe any sensitivity analyses	NA
**Results**			

For peer review only

Participants	13*	(a) Report numbers of individuals at each stage of study—eg numbers potentially eligible, examined for eligibility, confirmed eligible, included in the study, completing follow-up, and analysed	NA
		(b) Give reasons for non-participation at each stage	NA
		(c) Consider use of a flow diagram	NA
Descriptive data	14*	(a) Give characteristics of study participants (eg demographic, clinical, social) and information on exposures and potential confounders	9-10
		(b) Indicate number of participants with missing data for each variable of interest	NA
		(c) Summarise follow-up time (eg, average and total amount)	NA
Outcome data	15*	Report numbers of outcome events or summary measures over time	
Main results	16	(*a*) Give unadjusted estimates and, if applicable, confounder-adjusted estimates and their precision (eg, 95% confidence interval). Make clear which confounders were adjusted for and why they were included	11-12
		(*b*) Report category boundaries when continuous variables were categorized	NA
		(*c*) If relevant, consider translating estimates of relative risk into absolute risk for a meaningful time period	11
Other analyses	17	Report other analyses done—eg analyses of subgroups and interactions, and sensitivity analyses	NA
**Discussion**			
Key results	18	Summarise key results with reference to study objectives	13
**Limitations**			
Interpretation	20	Give a cautious overall interpretation of results considering objectives, limitations, multiplicity of analyses, results from similar studies, and other relevant evidence	13-14
Generalisability	21	Discuss the generalisability (external validity) of the study results	13-14
**Other information**			
Funding	22	Give the source of funding and the role of the funders for the present study and, if applicable, for the original study on which the present article is based	14

*Give information separately for cases and controls in case-control studies and, if applicable, for exposed and unexposed groups in cohort and cross-sectional studies.

Note: An Explanation and Elaboration article discusses each checklist item and gives methodological background and published examples of transparent reporting. The STROBE checklist is best used in conjunction with this article (freely available on the Web sites of PLoS Medicine at http://www.plosmedicine.org/, Annals of Internal Medicine at http://www.annals.org/, and Epidemiology at http://www.epidem.com/). Information on the STROBE Initiative is available at www.strobe-statement.org.

For peer review only
